# Restirring and Reheating Effects on Microstructural Evolution of Al–Zn–Mg–Cu Alloy during Underwater Friction Stir Additive Manufacturing

**DOI:** 10.3390/ma15113804

**Published:** 2022-05-26

**Authors:** Ying Li, Changshu He, Jingxun Wei, Zhiqiang Zhang, Ni Tian, Gaowu Qin, Xiang Zhao

**Affiliations:** 1 School of Materials Science & Engineering, Northeastern University, Shenyang 110819, China; liying3273@163.com (Y.L.); jingxunwei@foxmail.com (J.W.); lnkdzzq@126.com (Z.Z.); tiann@atm.neu.edu.cn (N.T.); qingw@smm.neu.edu.cn (G.Q.); zhaox@mail.neu.edu.cn (X.Z.); 2 Key Laboratory for Anisotropy and Texture of Materials, Northeastern University, Shenyang 110819, China; 3 Research Center for Metallic Wires, Northeastern University, Shenyang 110819, China

**Keywords:** friction stir additive manufacturing, Al–Zn–Mg–Cu alloy, microstructure, texture evolution, precipitation

## Abstract

Friction stir additive manufacturing (FSAM) can be potentially used for fabricating high-performance components owing to its advantages of solid-state processing. However, the inhomogeneous microstructures and mechanical properties of the build attributed to the complex process involving restirring and reheating deserve attention. This study is based on the previous research of the underwater FSAMed 7A04 aluminum alloy and adopts a quasi in situ experimental method, i.e., after each pass of the underwater FSAM, samples were taken from the build for microstructural observation to investigate the restirring and reheating effects on microstructural evolution during the underwater FSAM. Fine-grain microstructures were formed in the stir zone during the single-pass underwater FSAM. After restirring, the grain size at the bottom of the overlapping region decreased from 1.97 to 0.87 μm, the recrystallization degree reduced from 74.0% to 29.8%, and the initial random texture transformed into a strong shear texture composed of the C {110}<$1\bar{1}0$>. After reheating, static recrystallization occurred in the regions close to the new additive zones, increasing the grain size and recrystallization degree. This study not only revealed the microstructural evolution during the underwater FSAM but also provided a guideline for further optimization of the mechanical properties of the Al–Zn–Mg–Cu alloy build.

## 1. Introduction

Friction stir additive manufacturing (FSAM) is a new solid-state additive manufacturing technology [1]. Its basic working principle is similar to that of friction stir lap welding (FSLW). A non-consumable rotating tool is inserted into a set of overlapping plates with axial pressure, and the subsequent FSLW is conducted in the predefined directions to obtain the desired build [2,3]. During the FSAM, fine microstructures with equiaxed grains are obtained as a result of the severe plastic deformation and the corresponding dynamic recrystallization (DRX) [4,5]. Furthermore, in contrast to the fusion-based additive manufacturing, because FSAM is performed in the solid state without melting, no melting- and solidification-related defects, such as porosity, cracks and segregation, can be observed in the components [6,7]. Hence, the FSAM can be potentially used for fabricating high-performance components. It has been proven that the WE43 magnesium alloy build fabricated via the FSAM exhibited a high ultimate tensile strength (~400 MPa) and considerable ductility (17%) after an aging treatment [8]. However, on account of the characteristics of the layer-by-layer additive process, restirring and reheating occur in the FSAM process [9]. Different layers of the fabricated build experience different thermal cycles and various degrees of plastic deformation, resulting in the inhomogeneous microstructure and mechanical properties in the build [10].

As the build height increases, the closer a layer is to the bottom of the build, the more thermal cycles it experiences. For heat-treatable aluminum alloy builds, the sizes of grains and precipitates increased from the top to the bottom because of the multipass thermal cycles and the longtime static annealing. This resulted in a deterioration in the mechanical properties from the top to the bottom, leading to a macroscale softening phenomenon [11,12].

In addition to the different thermal exposures, the material flow at different layers of the build is disparate, owing to the different material flow governing mechanisms [13]. It has been pre-established that the material flow at the top and bottom regions of the stir zone during the friction stir welding (FSW) is mainly driven by the tool shoulder and the tool pin, respectively [14]. The main heat to plasticize the material is supplied by the frictional contact between the tool shoulder and the workpiece [15,16]. Therefore, a decrease in the deformation temperature and strain rate generally occurred from the top to the bottom of the stir zone [17,18]. This gradient of the deformation temperature and strain rate resulted in differences in grain size, dislocation density, recrystallization, and precipitation in different regions of the stir zone [19,20,21,22,23]. Additionally, during the deformation process, the shear deformation modes of the shoulder-driven and pin-driven are different, resulting in various texture components in the stir zone [24,25,26]. If the strain gradually decreases and the deformation is insufficient, the C-type texture replaces the B-/$\bar{B}$-type texture. Therefore, the C-type texture usually dominates the bottom region of the stir zone [27]. In contrast to the FSW, restirring occurred in the FSAM results in highly complex material flow features [28,29,30]. In particular, the adjacent stir zones overlap to form an overlapping region. The material in the non-overlapping region undergoes a single-pass stirring, and its flow is usually driven by the tool pin. Meanwhile, the material in the overlapping region experiences two-pass stirrings [31,32]. The material flow is first governed by the tool shoulder or the tool pin and then by the tool pin in the overlapping region. In the fabricated build, the macroscopic view of the transverse section reveals that the non-overlapping and overlapping regions appear alternately along the building direction. Accordingly, the pin-driven, shoulder- and pin-driven, and pin- and pin-driven material flow changes periodically along the building direction.

The periodic FSAM process results in periodic changes in the microstructure and in the mechanical properties of the build along the building direction [8,12,33,34,35,36]. The 5083-O aluminum alloy fabricated via the FSAM showed that the increased microhardness appeared at the bottom of each overlapping region, which may be related to the fine equiaxed grains formed by the restirring and thus fine-grain strengthening [33]. Conversely, Lu et al. [35] found that the microhardness was reduced at the bottom of each overlapping region of the 2050 aluminum alloy and did not recover after aging. The same phenomenon was observed in the 7075 [12] and 7A04 [36] aluminum alloys. A three-pass 7A04 aluminum alloy was fabricated by the underwater FSAM in our previous research [36]. The results indicated that underwater FSAM could effectively suppress the macroscale softening of the Al–Zn–Mg–Cu alloy from the top to the bottom. However, local softening, i.e., a low-hardness region at the bottom of each overlapping region that periodically exists along the building direction, occurred in the as-fabricated build and became more visible in the aged build. The main reason for the decrease in the mechanical properties and in the aging strengthening ability was the high density of the Mg(ZnAlCu)_2_ phase precipitation induced by the fine grains and the high density of subgrains and dislocations in this region.

In a practical additive manufacturing production, multipass or multilayered addition is also frequently used [37]. During such a process, the thermal history and material flow are complex, owing to restirring and reheating. Therefore, it is of practical importance to understand the effect of the current additive on the previous layers of the build and to reveal the microstructural evolution during the FSAM. However, most of the previous studies selected characteristic locations for microstructural observation and performance tests after completing the build. The effect of the process history on the microstructures and the mechanical properties was not investigated, especially for the underwater FSAM.

Therefore, this study is based on the previous research of the underwater FSAMed 7A04 aluminum alloy and adopts a quasi in situ experimental method, i.e., after each pass of the underwater FSAM, the samples are taken from the build for microstructural observation and microhardness testing to investigate the effects of restirring and reheating on grain size, recrystallization, local texture, and precipitation of the previous layers during the subsequent processing. This study not only reveals the microstructural evolution during the underwater FSAM but also provides a useful guideline for controlling the microscale softening of the underwater FSAM in the Al–Zn–Mg–Cu alloys for further optimization of the mechanical properties of the build.

## 2. Materials and Methods

**Figure 1:**
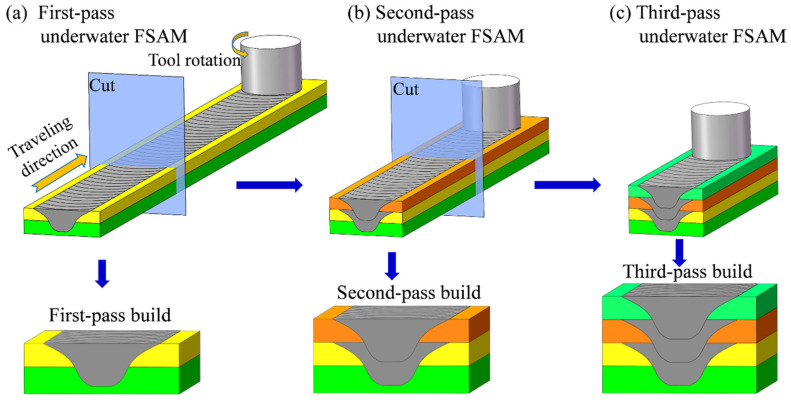
Schematics of (**a**) first-pass underwater friction stir additive manufacturing (FSAM); (**b**) second-pass underwater FSAM; and (**c**) third-pass underwater FSAM.

In this study, the 3.5 mm thick rolled plates of 7A04-T6 (Al–6Zn–2.6Mg–1.5Cu) aluminum alloy were used to conduct an underwater FSAM with a 700 r/min rotation speed and an 160 mm/min traveling speed. After each pass of the underwater FSAM, samples were taken from the build for microstructural observation and microhardness test. The detailed process is shown in Figure 1. First, two layers with a size of 300 × 25 × 3.5 mm were used for the first-pass underwater FSAM (shown in Figure 1a); after the first-pass underwater FSAM, the first third of the first-pass build was cut off for microhardness test and microstructural observation. A new layer was added on the first-pass build for the second-pass underwater FSAM, and the first third part of the second-pass build was cut after the completion of this process (shown in Figure 1b). Finally, the third-pass underwater FSAM was performed (shown in Figure 1c). As the periodic change of the mechanical properties has been found along the building direction after the three-pass FSAM in our previous study [36], investigating the microstructural characteristics during the three-pass FSAM is sufficient to reveal the microstructural evolution during the underwater FSAM in the Al–Zn–Mg–Cu alloy.

The microhardness distributions on the cross section of each stage build in the as-fabricated state and the artificial aging state (at 120 °C for 24 h) were obtained after the three-pass underwater FSAM using a Vickers microhardness tester (401-MVD, Wolpert Wilson Instruments) with a 100 g load and a microindentation grid with 0.5 × 0.5 mm indentation spacing.

To investigate the grain features, local texture, and precipitation evolution during the underwater FSAM, microstructural observation positions were selected based on the distribution characteristics of the microhardness. The grain features and local texture were investigated by electron backscatter diffraction (EBSD, ZEISS Gemini SEM 300). The samples were electropolished in a solution of 25 vol.% nitric acid and 75 vol.% methanol for 90 s at temperatures ranging from −30 to −25 °C and at a voltage of 15 V. The EBSD results were analyzed using the Channel 5 software (Oxford Instruments, Abingdon, UK). The grain orientation maps, the recrystallization, pole figures (PFs), and orientation distribution function (ODF) were generated from the EBSD data. To eliminate spurious boundaries caused by orientation noise, the minimum boundary disorientation was set to 2°. The grain boundaries in the range 2–15° were defined as low-angle grain boundaries (LAGBs), and those higher than 15° were defined as high-angle grain boundaries (HAGBs). In addition, the EBSD recrystallized fraction components, such as recrystallized, substructured, and deformed grains, were measured and calculated by the internal average misorientation angle within each grain [38]. The calculated internal average misorientation angle was compared with a minimum misorientation angle (2°) to define a subgrain. If the average angle in a grain exceeds 2°, the grain is classified as the “deformed.” If the grain consists of subgrains whose internal misorientation is less than 2° but the misorientation from subgrain to subgrain is more than 2°, the grain is classified as the “substructured.” The remaining grains are classified as the “recrystallized” [39].

The characteristics of the secondary phase particles in the as-fabricated state were analyzed by scanning transmission electron microscopy (STEM, JEOL JEM-2100F) combined with the energy dispersive X-ray spectroscopy (EDS). The samples were prepared by twin-jet thinning in a solution of 25 vol.% nitric acid and 75 vol.% methanol at temperatures ranging from −30 to −25 °C and at a voltage of 12 V.

## 3. Results

### 3.1. Microhardness Mapping

**Figure 2:**
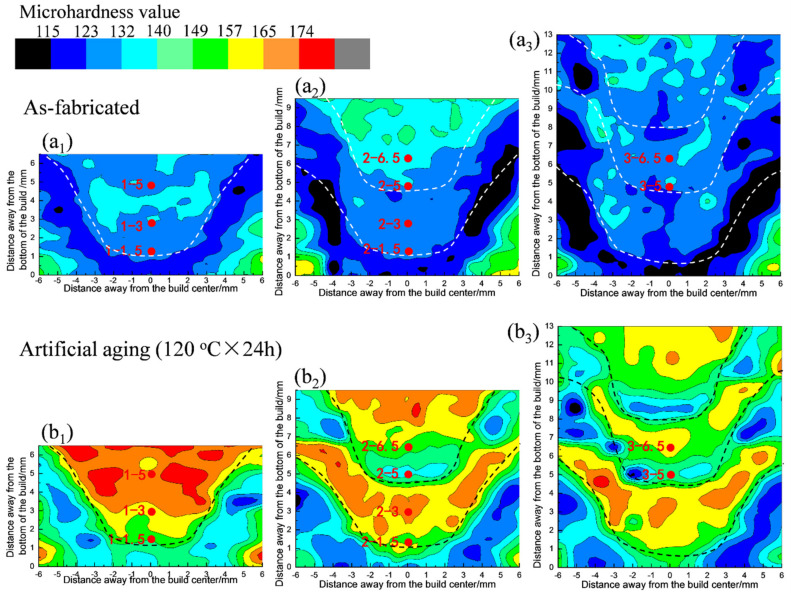
Microhardness map tested on the cross section of the (**a**) as-fabricated and (**b**) artificially aged states (120 °C for 24 h): microhardness maps of (**a_1_**,**b_1_**) first-pass build, (**a_2_**,**b_2_**) second-pass build, and (**a_3_**,**b_3_**) third-pass build. The dashed lines outline stir zones. The red dots mark the places for microstructure observation and the numbers indicate the distance in mm to the bottom of the build.

Figure 2 presents the microhardness distribution on the cross section of each stage build fabricated by the first-, second-, and third-pass underwater FSAM. The white and black lines in Figure 2 outline the boundaries of the stir zones. As shown in Figure 2a_1_–a_3_, the microhardness variation in the as-fabricated state was not obvious. However, after the aging treatment (120 °C for 24 h) (Figure 2b_1_–b_3_), there were clear local differences in microhardness. In the first-pass build, the microhardness decreased from the top to the bottom region (Figure 2b_1_). After the second-pass underwater FSAM, the microhardness of the restirred region (overlapping region) decreased. Moreover, the decrease in microhardness at the bottom of the overlapping region was more obvious than those in the other regions (Figure 2b_2_). Meanwhile, the microhardness of the regions that underwent reheating had no obvious change because the FSAM was conducted underwater. After the third-pass underwater FSAM, the microhardness of the regions that underwent only sequential reheating did not change obviously. The microhardness of the new overlapping region also decreased, similar to the second-pass underwater FSAM.

The microhardness distribution of the heat-treatable aluminum alloy build was mainly determined by the evolution of the strengthening precipitates, which was further influenced by the plastic deformation, thermal cycling and the original α-Al matrix features (including grain size, dislocation/substructure features, etc.) [40]. The local variation in microhardness of the artificially aged builds reflect that the microstructures of the original α-Al matrix of the fabricated build were varied. The varied microstructure affects the precipitation behavior during the aging process and in turn influencing the mechanical properties of the build. Thus, the observation positions of the local microstructure were determined according to the characteristics of the microhardness distribution on the cross section of the different stage builds in the artificially aged state. However, the samples of the local microstructures were taken from the as-fabricated builds (marked with the red dots in Figure 2a_1_–a_3_) to avoid the effect of artificial aging on the evolution of the initial microstructure during the underwater FSAM.

### 3.2. Microstructure

**Figure 3:**
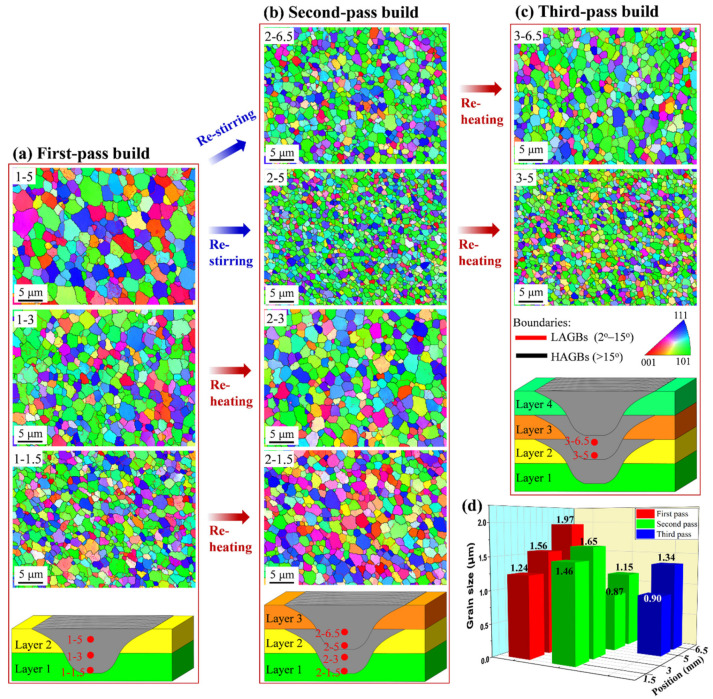
SEM-EBSD micrographs and stir zone illustrations of the (**a**) first-, (**b**) second-, and (**c**) third-pass builds; (**d**) average grain sizes in specified positions (**a**–**c**) are marked with red dots.

Figure 3 shows the grain morphology and grain size in different regions of the first-, second-, and third-pass builds. It is seen that after the first-pass underwater FSAM, the grains in all the selected regions (Figure 3a) are in more or less fine equiaxed form that are different from those of the initially cold-rolled state, indicating that dynamic recrystallization occurred in the stir zone due to the severe plastic deformation and the adiabatic heating resulting from the deformation [41]. The average grain sizes of the top (position 1-5), middle (position 1-3) and bottom (position 1-1.5) regions were 1.97, 1.56 and 1.24 µm, respectively (Figure 3a,d)). The gradual decrease in the average grain size from the top to the bottom regions of the first-pass build can mainly be attributed to the decrease in the deformation temperature and strain rate. 

When adding a new layer to continue the second-pass underwater FSAM process (Figure 3b), the material in the overlapping region (positions 2-6.5 and 2-5) experienced restirring, and the grain sizes in positions 2-6.5 and 2-5 decreased to 1.15 and 0.87 µm, respectively. The grain refinement was more significant at the bottom of the overlapping region (position 2-5), which was mainly attributed to the low deformation temperature and the low strain rate. Moreover, the second phase particles precipitated along the grain boundaries could further inhibit grain growth. The detailed particle features are analyzed in Section 3.4. Meanwhile, the material in the non-overlapping region underwent reheating. As shown in Figure 3b,d, the grain sizes in positions 2-3 and 2-1.5 were 1.65 and 1.46 µm, respectively, which were slightly higher than those in positions 1-3 (1.56 µm) and 1-1.5 (1.24 µm) of the first-pass build. However, grain growth was not obvious because the FSAM was conducted underwater. 

After the third-pass underwater FSAM (Figure 3c), the overlapping region experienced reheating. The grain size in position 3-6.5, which close to the new additive zone, slightly increased from 1.15 to 1.34 µm, whereas the grain size at position 3-5 (~0.90 µm) did not change obviously. This was also because the water cooling applied in the FSAM could weaken the subsequent thermal effect on the previous additive layers.

**Figure 4:**
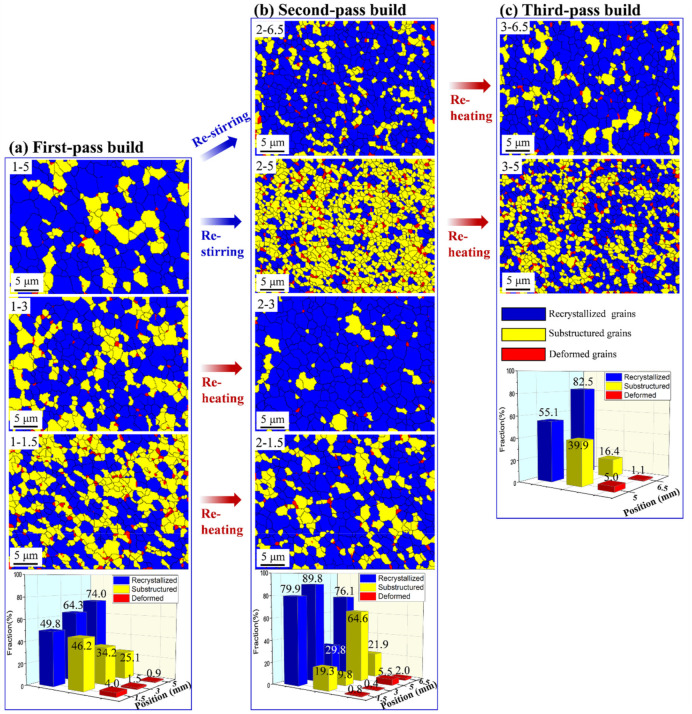
SEM-EBSD recrystallization fraction micrographs of the (**a**) first-, (**b**) second-, and (**c**) third-pass builds together with the histograms of recrystallized, substructured and deformed fractions. The specified positions of the microstructural observation are shown in Figure 3a–c, marked with the red dots.

Figure 3 also shows that these equiaxed grains are partially surrounded by HAGBs and partially by LAGBs. The LAGBs were usually formed by the continuous dislocation accumulation and rearrangement during dynamic recovery (DRV) accompanying the deformation [42,43] and subdivided the original large elongated initially cold-rolled grains. The high density of intragranular LAGBs indicated that the DRX was insufficient. Figure 4 presents the recrystallized, substructured, and deformed fractions in different regions of the first-, second-, and third-pass builds. As shown in Figure 4a, the recrystallized fractions of the top (position 1-5), middle (position 1-3), and bottom (position 1-1.5) regions after the first-pass underwater FSAM were 74.0%, 64.3%, and 49.8%, respectively. The recrystallized fraction decreased from the top to the bottom regions. On the contrary, the substructured and deformed fraction increased, which was mainly attributed to the decreasing deformation temperature and strain rate from the top to the bottom regions. The low deformation temperature and strain rate suppressed the DRX progress in the bottom region by inhibiting the transformation of LAGBs into HAGBs.

In the second-pass build, the degrees of recrystallization at different positions of the restirred region varied. As shown in Figure 4b, the recrystallized area fraction in position 2-6.5 was 76.1%, which was similar to that in position 1-5 (~74%). However, the recrystallized area fraction in position 2-5 decreased from 74.0% to 29.8%. The decrease in recrystallization at the bottom of the restirred region indicated that the DRX process was suppressed by decreasing the dislocation moving in the low deformation temperature and low strain rate condition. Meanwhile, the moving dislocations were subjected to being pinned by the second phase particles at the grain boundaries or the subgrain boundaries [44]. This would further suppress the DRX process, and lead to the increase in the substructured and deformed fractions.

After reheating during the second-pass underwater FSAM, the recrystallized area fractions in positions 2-3 and 2-1.5 increased to 89.8% and 79.9%, respectively. A similar phenomenon also occurred in the third-pass build. As shown in Figure 4c, the recrystallized area fractions in positions 3-6.5 and 3-5, which experienced reheating during the third-pass underwater FSAM, increased to 82.5% and 55.1%, respectively. The increase in the recrystallization should be primarily governed by static recrystallization (SRX) that occurred during reheating.

### 3.3. Local Texture

**Figure 5:**
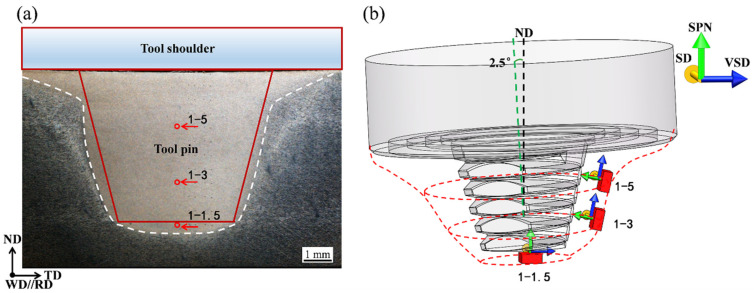
(**a**) Schematic of positions 1-5, 1-3, and 1-1.5 with respect to the stir tool; (**b**) shear surface of the top (position 1-5), middle (position 1-3), and bottom (position 1-1.5) regions.

In addition to the difference of microstructures, the local textures vary throughout the thickness of the stir zone owing to the different shear deformation modes. The rotation of the stir tool directly affects the crystallographic orientation of the material [45]. As shown in Figure 5a, the material flow in the top (position 1-5) and middle (position 1-3) regions are mainly driven by tool pin rotation, however, the material flow in the bottom (position 1-1.5) region is mainly driven by the end of the tool pin. The schematic diagrams of the shear surfaces in the top, middle, and bottom regions are illustrated in Figure 5b. The shear direction (SD) is tangential to the shear surface, and the shear plane normal (SPN) is perpendicular to that surface.

**Figure 6:**
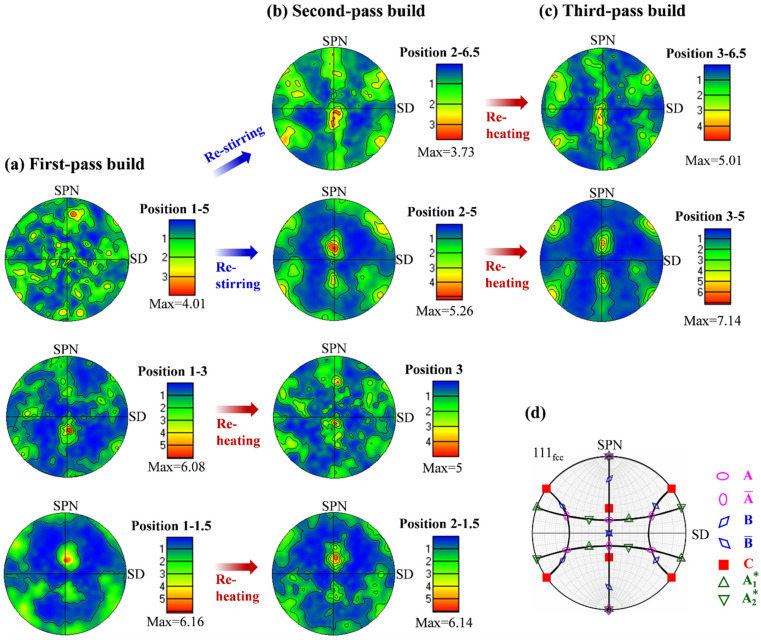
{111} Pole figures (PFs) in different regions of the (**a**) first-, (**b**) second-, and (**c**) third-pass builds; (**d**) ideal orientations of face-centered cubic metals under simple shear [46,47]. The specified positions of the PFs are shown in Figure 3a–c, marked with red dots.

**Figure 7:**
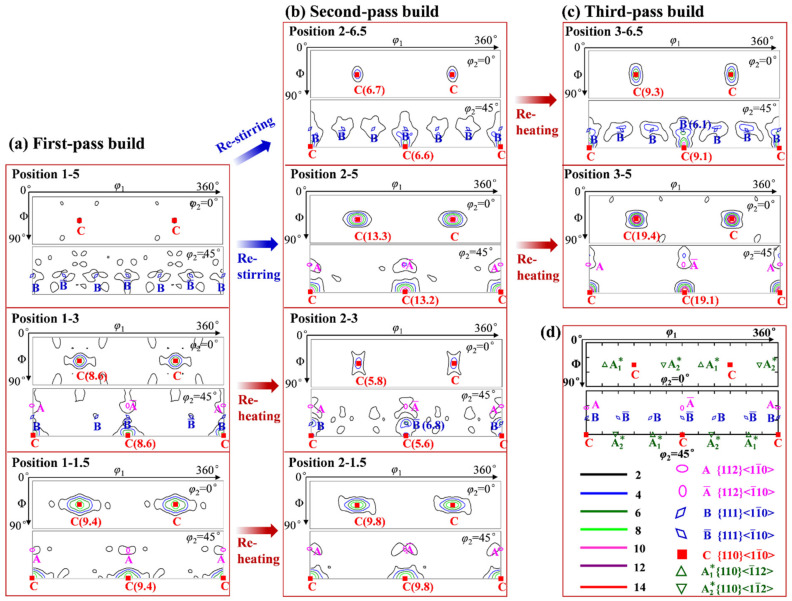
*φ*_2_ = 0° and *φ*_2_ = 45° sections of orientation distribution function (ODF) in different regions of the (**a**) first-, (**b**) second-, and (**c**) third-pass builds; (**d**) ideal orientations of face-centered cubic metals under simple shear in the *φ*_2_ = 0° and *φ*_2_ = 45° ODF sections [47]. The specified positions of the ODFs are shown in Figure 3a–c, marked with red dots.

Figure 6 and Figure 7 show the {111} pole figures (PFs) and the corresponding orientation distribution functions (ODFs) (*φ*_2_ = 0° and 45° sections) of different regions in the first-, second-, and third-pass builds. In the first-pass build (Figure 6a and Figure 7a), the texture is nearly random in the top region, and it gradually evolved into a characteristic one from the middle to the bottom region with enhanced intensities. According to the ideal shear texture components of the face-centered cubic (FCC) metals [46,47] shown in Figure 6d ({111} PF) and Figure 7d (*φ*_2_ = 0° and 45° ODF sections), the texture is characterized by a strong C-type shear component ({110}<$1\bar{1}0$>) and a very weak A/$\bar{A}$-type shear component ({112}<110>) and B/$\bar{B}$-type component ({111}<110>) in position 1-3. In position 1-1.5, the shear texture of C {110}<$1\bar{1}0$> was dominant. In the second-pass build (Figure 6b and Figure 7b), after the restirring, a weak component of respective C and B/$\bar{B}$-type components appeared in position 2-6.5. Meanwhile, the texture in position 2-5 evolved into a strong C component and a weak A/$\bar{A}$-type component from the initial random texture in position 1-5. However, after reheating from the second-pass underwater FSAM, the texture features in positions 2-3 and 2-1.5 did not obviously change compared with those of the first-pass build. The only change that happened in position 2-3 was that the intensity of the C component decreased and that of B/$\bar{B}$ component slightly strengthened. After the third-pass underwater FSAM, the types of texture components in positions 3-6.5 and 3-5 after reheating did not change (Figure 6c and Figure 7c), but the intensities of the corresponding components varied, with a strengthening of all the components.

### 3.4. Secondary Phase Particles

**Figure 8:**
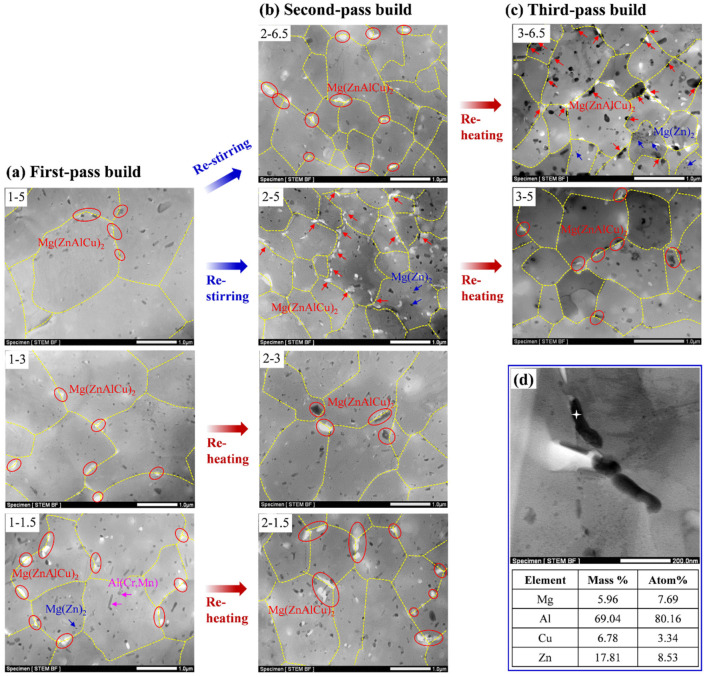
Scanning transmission electron microscopy (STEM) images of the (**a**) first-, (**b**) second-, and (**c**) third-pass builds; and (**d**) STEM–EDS result of the phase along the grain boundary. The specified positions of the STEM images are shown in Figure 3a–c, marked with red dots.

The STEM images of the various regions of the first-, second-, and third-pass builds are presented in Figure 8a–c. Some large precipitates (0.2–0.6 µm) were observed along the grain boundaries. EDS analysis result shows that these precipitates contain Al, Zn, Mg, and Cu (Figure 8d). By combining the morphology, size, and chemical composition of these particles reported in the previous studies [48], the large particle was recognized as the Mg(ZnAlCu)_2_ phase, which mainly deteriorated the mechanical properties of the build. The grain boundaries, subgrain boundaries, and dislocations are rapid diffusion channels for solute atoms [49]. During the high-temperature deformation process, Zn, Mg, and Cu atoms in the solid solution tended to segregate at grain boundaries and formed the Mg(ZnAlCu)_2_ phase, and then grew rapidly and finally exhibited a large size [50]. Moreover, these large Mg(ZnAlCu)_2_ particles are easily shedded during the sample preparation by electropolishing, and thus, some small holes caused by the shedding of the large particles were observed along the grain boundaries. Figure 8a–c show that the number of large Mg(ZnAlCu)_2_ particles is different in various regions of the multipass builds. In the first-pass build, the number of grain boundary Mg(ZnAlCu)_2_ particles increased from the top (position 1-5) to the bottom (position 1-1.5). After restirring, the number of grain boundary Mg(ZnAlCu)_2_ increased in positions 2-6.5 and 2-5 in the second-pass build compared to that in position 1-5 of the first-pass build. Moreover, the increase in position 2-5 was more obvious. After the reheating, the number of Mg(ZnAlCu)_2_ particles in positions 2-3 and 2-1.5 did not change obviously, compared to those in positions 1-3 and 1-1.5. The same features were shown in positions 3-5 and 3-6.5, which underwent reheating during the third-pass underwater FSAM.

From the abovementioned STEM observation and the microstructures in various regions of the builds, the grain size is a key factor that affects the number of grain boundary Mg(ZnAlCu)_2_ particles. The smaller the grain is, the more Mg(ZnAlCu)_2_ particles precipitate along the grain boundary. During underwater FSAM, restirring significantly refined the grain size in the overlapping region of the build and promoted the precipitation of Mg(ZnAlCu)_2_ particles along the grain boundaries. These grain boundary Mg(ZnAlCu)_2_ particles could prevent grain growth by pinning the grain boundaries and subgrain boundaries. Additionally, these precipitates also suppressed the DRX process through hindering dislocation movement. The abovementioned phenomena were more obvious in the bottom of the overlapping region. However, the reheating effects on dissolution, reprecipitation, and growth of the Mg(ZnAlCu)_2_ phase were not obvious owing to water cooling.

In addition to the grain boundary Mg(ZnAlCu)_2_ phase, Al(Cr,Mn) and MgZn_2_ phases were observed in the grain interiors. The difference in the number of Al(Cr,Mn) particles across various regions was not obvious; however, the MgZn_2_ particles, which preferentially precipitated along the dislocations and substructures, increased at the bottom of the overlapping regions (positions 2-5 and 3-5).

## 4. Discussion

**Figure 9:**
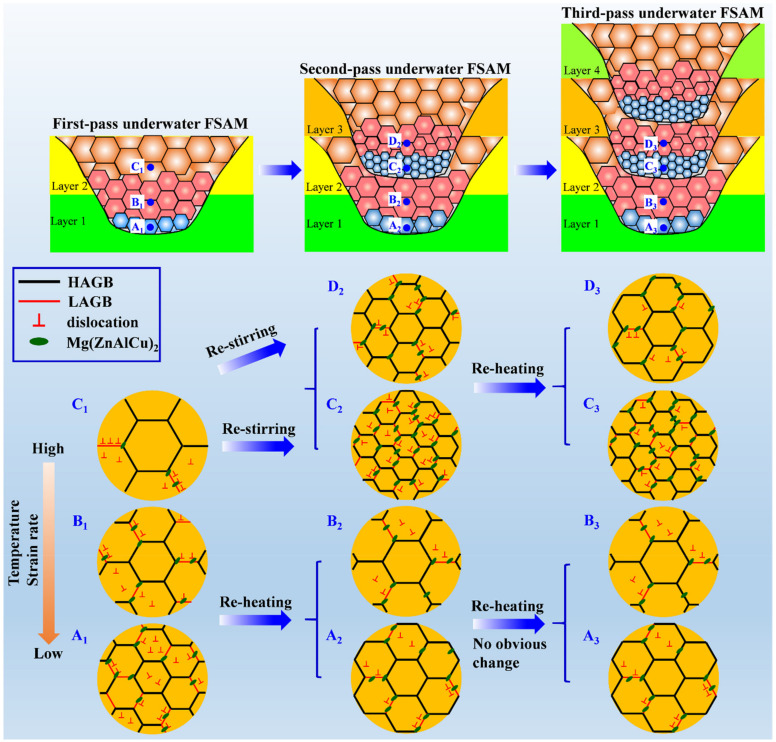
Schematic of microstructural evolution during underwater FSAM. The specified positions of the microstructures are marked with blue dots and letters (A_1_, A_2_, A_3_, B_1_, B_2_, B_3_, C_1_, C_2_, C_3_, D_2_, D_3_).

The strengthening of the heat-treatable aluminum alloy builds primarily depends on the precipitate evolution [51]. The precipitation behavior is influenced not only by the deformation temperature, strain rate, and cooling rate [52,53], but also by the characteristics of the α-Al matrix, including grain size, density of dislocations and substructures [54]. Figure 9 presents the schematic of the microstructural evolution during underwater FSAM in 7A04 aluminum alloy. The effects of restirring and reheating during the underwater FSAM on grain size, recrystallization, local texture, and precipitation behavior were analyzed, and the influence of microstructural evolution on the mechanical properties of the build was further evaluated.

During the single-pass underwater FSAM (i.e., first-pass underwater FSAM), fine equiaxed grains were formed in the stir zone due to the occurrence of DRX. From the top (position C_1_) to the bottom (position A_1_) region of the stir zone, as the deformation temperature and strain rate gradually decreased, the grain size decreased. Meanwhile, the lower deformation temperature and strain rate also decreased the movement of dislocations, thereby inhibiting the transition from LAGBs to HAGBs during DRX, leading to lower recrystallization degree and higher density of dislocations and subgrains in the matrix of the bottom region than those of other regions. Furthermore, the PFs and ODF results showed that the strong shear texture of C component appeared at the bottom of the stir zone (Figure 6 and Figure 7). Materials with C shear texture have higher dislocation density and stored energy than materials with A/$\bar{A}$, $A_{1}^{*}$/$A_{2}^{*}$, and B/$\bar{B}$ shear components [55]. These fine grains and the high density of dislocations and substructures at the bottom of the stir zone promoted the precipitation of the coarse Mg(ZnAlCu)_2_ phase, resulting in a decrease in the aging strengthening ability and mechanical properties in this region.

After adding a new layer to continue the underwater FSAM (i.e., second-pass underwater FSAM), restirring and reheating occurred in the build. After restirring, the grain size in the overlapping region (positions C_2_ and D_2_) significantly reduced compared with that of the top region of the first-pass build (position C_1_). In particular, the grain size at the bottom of the overlapping region (position C_2_) reduced from the original 1.97 to 0.87 μm, the recrystallization degree reduced from 74.0% to 29.8%, and the texture changed from the original random texture to a strong C shear one. The abovementioned changes at the bottom of the overlapping region were correlated with the original grain size, grain boundary precipitates, and the deformation temperature and strain rate during restirring. Fine grains were already formed in the previous process. Thus, the original fine grains were further separated into individual grains by LAGBs during the subsequent restirring process. Furthermore, the low deformation temperature and strain rate at the bottom of the overlapping region inhibited the grain growth. Therefore, the finer grains formed. In addition, the low deformation temperature and strain rate in this region also suppressed the DRX process, leading to a high density of substructures preserved during the restirring process. These finer grains and higher density of substructures intensified the precipitation of the coarse Mg(ZnAlCu)_2_ phase. These coarse precipitates not only inversely hindered the grain growth, but also suppressed the DRX process in the low deformation temperature and strain rate condition and resulted in a strong C shear texture appeared in this region. Moreover, these coarse Mg(ZnAlCu)_2_ phases further reduced the aging strengthening ability and decreased the mechanical properties of the bottom of the overlapping region. As for the top of the overlapping region (position D_2_), which also underwent restirring, although the grain size reduced to 1.15 μm, the recrystallization degree and texture characteristics did not change significantly. This was attributed to the higher deformation temperature and strain rate at the top of the overlapping region than that at the bottom, which induced DRX during the restirring process. The number of dislocations and substructures inside the grains was smaller than that at the bottom of the overlapping region. Thus, fewer Mg(ZnAlCu)_2_ phases precipitated along the grain boundaries, and the aging strengthening ability and mechanical properties increased. 

In contrast to positions A_1_ and B_1_ of the first-pass underwater FSAM, positions A_2_ and B_2_ underwent reheating of the new additive and SRX occurred. The number of dislocations and substructures in the grains reduced, and the recrystallization degree increased. However, because FSAM was conducted underwater, the reheating of the new additive was limited; therefore, the grain size was less increased, and the texture characteristics have not changed significantly. In addition, the limited thermal cycling effect is not enough to dissolve or coarsen the coarse Mg(ZnAlCu)_2_ phase that has precipitated along the grain boundaries, and thus, the mechanical properties of these regions are not significantly changed.

By adding another layer (i.e., third-pass underwater FSAM), both the overlapping (positions C_3_ and D_3_) and non-overlapping (positions A_3_ and B_3_) regions were subjected to the reheating of the new additive. Positions C_3_ and D_3_, which are close to the new additive zone, underwent SRX after reheating. The grain size increased slightly, the density of dislocations and substructures decreased, and the recrystallization degree increased significantly, but the local textures and Mg(ZnAlCu)_2_ particles did not change obviously. As for positions A_3_ and B_3_ which are away from the new additive stir zone, there were no obvious changes in the grain size, recrystallization degree, local texture, and precipitation because of the limited thermal cycle of the underwater FSAM. Therefore, the aging strengthening ability and mechanical properties have no obvious changes under thermal exposure.

As the abovementioned analysis, restirring occurred in the state of low deformation temperature and low strain rate significantly decreased the grain size and suppressed the recrystallization, leading to the incompletely recrystallized fine structures and high-density substructures at the bottom of the overlapping region. The refined grains and high-density substructures promoted the precipitation of the Mg(ZnAlCu)_2_ phase along grain boundaries, directly leading to low-degree supersaturation and decreasing the aging-strengthening ability. Conversely, they also accelerated the precipitation of the Mg(Zn)_2_ phase in the subsequent artificial aging process. It is easy to cause overaging if the traditional aging treatment (120 °C for 24 h) is still used as the post-aging. These reasons co-induced the local softening appeared in Al–Zn–Mg–Cu alloy build after aging treatment. Therefore, future investigations will focus on the post-aging effect on the microstructures and mechanical properties of the build and selecting a suitable aging treatment to control the local softening behavior.

## 5. Conclusions

The restirring and reheating effects of the underwater FSAM on microstructure, local texture, and precipitation of the 7A04 Al alloy build were investigated in this study. The primary conclusions can be drawn as follows:

(1) During the single-pass underwater FSAM, the grain size and recrystallization degree decreased from the top to the bottom region. Furthermore, the texture is nearly random in the top region, and it gradually evolved into a characteristic C {110}<$1\bar{1}0$ > component from the middle to the bottom region with enhanced intensities.
(2) After restirring during the subsequent process, the grain size at the bottom of the overlapping region decreased from 1.97 to 0.87 μm, the recrystallization degree reduced from 74.0% to 29.8%, and the initial random texture transformed into a strong shear texture composed of the C component.
(3) After reheating, the grain size and recrystallization degree in the regions close to the new additive zone slightly increased. However, the local texture and precipitation did not change obviously because of the limited thermal exposure during underwater FSAM. Additionally, reheating has no obvious effects on the microstructures of the regions away from the new additive zone.
(4) The refined grains and the high density of the substructures caused by the restirring at the bottom of the overlapping region promoted the precipitation of the Mg(ZnAlCu)_2_ phase along grain boundaries. However, the reheating effects on the dissolution, reprecipitation, and growth of the Mg(ZnAlCu)_2_ phase were not obvious because of water cooling.

## Data Availability

The raw/processed data required to reproduce these findings cannot be shared at this time as the data also comprise a part of an ongoing study.
